# A Systematic Investigation of the Effect of Action Observation Training and Motor Imagery Training on the Development of Mental Representation Structure and Skill Performance

**DOI:** 10.3389/fnhum.2017.00499

**Published:** 2017-10-17

**Authors:** Taeho Kim, Cornelia Frank, Thomas Schack

**Affiliations:** ^1^Neurocognition and Action-Biomechanics Research Group, Bielefeld University, Bielefeld, Germany; ^2^Cognitive Interaction Technology—Center of Excellence (CITEC), Bielefeld University, Bielefeld, Germany; ^3^Research Institute for Cognition and Robotics (CoR-Lab), Bielefeld University, Bielefeld, Germany

**Keywords:** motor learning, cognitive training, skill acquisition, golf putting, SDA-M, simulation theory

## Abstract

Action observation training and motor imagery training have independently been studied and considered as an effective training strategy for improving motor skill learning. However, comparative studies of the two training strategies are relatively few. The purpose of this study was to investigate the effects of action observation training and motor imagery training on the development of mental representation structure and golf putting performance as well as the relation between the changes in mental representation structure and skill performance during the early learning stage. Forty novices were randomly assigned to one of four groups: action observation training, motor imagery training, physical practice and no practice. The mental representation structure and putting performance were measured before and after 3 days of training, then after a 2-day retention period. The results showed that mental representation structure and the accuracy of the putting performance were improved over time through the two types of cognitive training (i.e., action observation training and motor imagery training). In addition, we found a significant positive correlation between changes in mental representation structure and skill performance for the action observation training group only. Taken together, these results suggest that both cognitive adaptations and skill improvement occur through the training of the two simulation states of action, and that perceptual-cognitive changes are associated with the change of skill performance for action observation training.

## Introduction

Motor learning means a relatively permanent change in the competence of skill performance, resulting from systematic and repeated practice (e.g., Magill, 2000). The learning of a motor skill is commonly attained via physical repetition of a skill before moving to a different motor skill (e.g., Coker, 2004). However, research has shown that cognitive training, such as motor imagery and action observation training, can also be applied effectively to facilitate skill learning, either alone, or combined with physical practice (e.g., Hodges and Williams, 2012).

Motor imagery refers to a dynamic state during which learners simulate specific motor actions mentally, without actual movement (e.g., Jeannerod, 1995; Decety, 1996). Furthermore, motor imagery training has been used as an effective means to facilitate motor learning and performance (e.g., Driskell et al., 1994; Murphy, 1994; Schack et al., 2014). Meta-analyses on this topic have reported that motor imagery training has a positive effect on motor performance, even though the degree of its effectiveness varies with the moderators, such as type of task, experience level of participants, duration of practice, and other factors (e.g., Driskell et al., 1994). In addition, motor imagery training was shown to be more effective compared to no practice, but was less effective than physical practice. Moreover, the combination has been proven to be as effective, or even more effective, than either motor imagery or physical practice alone (e.g., Hall et al., 1992; Liu et al., 2014; Bajaj et al., 2015). Thus, these findings show that motor imagery training can be an effective type of cognitive training as a complement to physical practice to enhance motor outcomes.

Action observation is an effective means of observational practice that has been considered extensively for enhancing motor learning and performance (e.g., Ste-Marie et al., 2012), as well as for modifying social behavior (e.g., Bandura, 1986). Research on action observation showed that action observation training benefits not only performance production variables like movement coordination pattern (e.g., Horn et al., 2007), but also performance outcome variables related to motor learning (e.g., Hayes et al., 2008). To assess the motor learning effect of action observation training, previous studies compared the effect of action observation training with the effect of physical practice or no practice. From this, action observation training was found to be superior to no practice (e.g., Kohl and Shea, 1992; Hayes et al., 2008). In addition, it was suggested that the combination of action observation training and physical practice provides more unique opportunities than either action observation or physical practice alone (e.g., Shea et al., 2000; Weeks and Anderson, 2000). These findings show that action observation training can be an effective type of cognitive training as a complement to physical practice to facilitate behavior outcomes.

Jeannerod (2001) has proposed that simulation states (S states) such as action observation and motor imagery are functionally equivalent to action execution, assuming that both are based on action representations encoded in the brain. This proposal has been supported by many brain-image studies, which showed that both action observation and motor imagery lead to the activation of motor-related brain areas (e.g., Lotze et al., 1999; Filimon et al., 2007; Lorey et al., 2013). These studies suggested that, to some extent, they make use of the same neural substrates as those involved during the execution of observed or imagined actions. Furthermore, many studies have reported that the neural representations for action observation and motor imagery are somewhat similar to those for motor execution (e.g., Clark et al., 2004; Filimon et al., 2007; Zabicki et al., 2017). For action observation, motor-related information, which is available through the visual system, is encoded into a type of mental representation in long-term memory for the organization of a future intended action (e.g., Holmes and Calmels, 2008; Ste-Marie et al., 2012). Motor imagery is required to consciously retrieve a stored mental representation in long-term memory (e.g., Jeannerod and Decety, 1995; Bandura, 1997; Soohoo et al., 2001; Wright et al., 2014). These indicate that mental representations, which play a key role in the control and organization of intended actions (e.g., Land et al., 2013), are involved during action observation and motor imagery.

Although motor imagery and action observation rely on a similar action representation, there is a difference between them in aspects of the mechanism of cognitive process. Motor imagery is a knowledge-driven cognitive process that is internally simulated based on information in long-term memory, without an external stimulus (e.g., Murphy, 1994; Soohoo et al., 2001; Holmes and Calmels, 2008; Helm et al., 2015). Instead, action observation is a percept-driven cognitive process that is externally guided by an external stimulus, such as a live demonstration or recorded video (e.g., Ram et al., 2007; Holmes and Calmels, 2008; Vogt et al., 2013). Therefore, motor imagery completely relies on mental representations stored in long-term memory to generate a motor image (e.g., Farah, 1984; Mulder et al., 2004; Frank et al., 2014; Schack et al., 2014). On the contrary, action observation is not dependent on the mental representation in long-term memory, since it completes a percept-driven process (e.g., Guillot and Collet, 2010; Wright et al., 2014); that is, visual information provided externally is held in working memory, and does not necessarily rely on representations stored in long-term memory. This implies that, despite the functional equivalence between action observation and motor imagery, the two simulation states (S-states) of action may not necessarily lead to the same effect in the improvement of the mental representation and performance.

The perceptual-cognitive perspective specifies that cognitive representations, which guide motor actions, are formed based on perceptual information (Zentgraf et al., 2009). In this sense, the perceptual-cognitive perspective on motor control addresses the idea that intended and executed motor actions are based on the mental representation of motor actions stored in long-term memory (e.g., Mechsner et al., 2001; Schack and Mechsner, 2006). From the cognitive action architecture approach (CAA-A; Schack, 2004; for reviews see e.g., Land et al., 2013), the organization of motor action for the execution is functionally guided by mental representations encoded in long-term memory. In other words, mental representations function as a cognitive reference for movement control. The framework of mental representation is composed of basic action concepts (BACs), which are identified as major representation units for complex actions (e.g., Schack, 2004; Schack and Mechsner, 2006). Based on the CAA-A, functional changes in the relation of BACs appear over the motor learning process within a conceptual framework in long-term memory (e.g., Bläsing et al., 2009; Schack and Ritter, 2009; Frank et al., 2013).

To investigate the role of mental representations in long-term memory, Schack and Mechsner (2006) compared the difference in mental representation structure according to different skill levels, employing the structural dimensional analysis of mental representation (SDA-M; for more details see Schack, 2012). According to the results, mental representation structures of high-level experts were not only well organized, but also corresponded to functional demands of the task. In contrast, mental representation structures of low-level players and novices were relatively less organized and less linked to the functional demands of the task. Similar results were also reported in some studies that examined the difference in mental representation structure with skill expertise using the SDA-M for diverse motor tasks (e.g., Schack and Hackfort, 2007; Bläsing et al., 2009; Velentzas et al., 2010). In addition, according to Frank et al. (2013), the mental representation structure of golf novices became functionally more organized with physical practice over time, whereas no practice did not cause any changes in mental representation structure. These findings suggest that mental representations serve as a basis for action control, adapting functionally over the course of motor learning.

Given that most motor skills include both physical and cognitive elements, not only physical practice but also cognitive training may lead to the development of mental representation. Recently, Frank et al. (2014) examined the effect of motor imagery training on mental representation structure and performance of novice golf players. The study showed that mental representation structure was functionally well organized particularly following motor imagery training. Moreover, in a recent study by Frank et al. (under review), it was shown that action observation training for golf beginners led to more functional mental representation structures along with performance improvement. These findings suggest that both motor imagery training and action observation training can enhance the functional adaption of task-specific mental representation in the early learning stage.

Taken together, motor imagery training and action observation training in themselves have a positive effect on the improvement of skill performance of novice learners in the early skill acquisition stage. Some studies have compared the differences in neurophysiologic and behavioral effects between motor imagery training and action observation training. Nevertheless, to date, no studies have compared how motor imagery and action observation training affect the formation process of mental representation in novices. Thus, in this study, we aimed at investigating the differences in the effects of the two types of cognitive training on the development of mental representation structure and the performance of a golf putting task. It was expected that motor imagery training and action observation training, as well as physical practice, would lead to functional changes of mental representation structure, along with performance improvement, compared to no practice (i.e., control group). Furthermore, it was expected that action observation training would be relatively more effective than motor imagery training in the development of mental representation, since it was predicted that novices have available limited representation structure of the golf putt in their long-term memory. Therefore, we expected that the allocation of new and well-structured motor information through action observation would lead to more elaborate representation and better performance than the simulation through motor imagery. In addition, based on the CAA-A, it was expected that the structured change of mental representation would be somewhat linked to performance improvement.

## Materials and Methods

### Participants

Forty participants from local university (18 males, 22 females; *M_age_* = 25.20, *SD* = 4.12) took part in this study. All participants were beginners with no prior experience in golf, and had normal or corrected-to-normal vision. They were randomly assigned into four groups, maintaining an equal group size: action observation training group (*n* = 10, *M*_age_ = 23.30, *SD* = 3.40, 4 males), motor imagery training group (*n* = 10, *M*_age_ = 26.50, *SD* = 3.87, 4 males), physical practice group (*n* = 10, *M*_age_ = 26.30, *SD* = 4.79, 4 males), and control group with no practice (*n* = 10, *M*_age_ = 24.70, *SD* = 4.06, 6 males). This study was carried out in accordance with the recommendations of the ethics committee of the University of Bielefeld (EUB) with written informed consent from all subjects. All subjects gave written informed consent in accordance with the Declaration of Helsinki. The protocol was approved by the ethics committee of the University of Bielefeld (EUB).

### Measurement

#### Mental Representation Structure

The SDA-M was used to evaluate the mental representation structure (e.g., Schack, 2012) of the golf putt. This method provides psychometric information on the structure of movement representation in long-term memory. More specifically, it is possible to investigate the status of the clustering and relations regarding BACs of a motor action through SDA-M. Thus, SDA-M has been employed as a reliable method to measure the mental representation structure of motor action (e.g., Schack and Mechsner, 2006; Bläsing et al., 2009; Frank et al., 2013). The following 16 BACs for golf putting that were developed by Frank et al. (2013) were applied in this study (see Table 1). From a functional and biomechanical perspective, each of the 16 BACs can be allocated to one of four movement phases: BAC 1–4 (preparation phase), BAC 5–7 (backswing phase), BAC 8–11 (forward swing phase), and BAC 12–16 (attenuation phase).

**Table 1:** Basic action concepts of the golf putt.

Movement phase	Number	Basic action concept (BAC)
Preparation	1	Shoulders parallel to target line
	2	Align club face square to target line
	3	Grip check
	4	Look to the hole
Backswing	5	Rotate shoulders away from the ball
	6	Keep arms-shoulder triangle
	7	Smooth transition
Forward swing	8	Rotate shoulders towards the ball
	9	Accelerate club
	10	Impact with the ball
	11	Club face square to target line at impact
Attenuation	12	Follow-through
	13	Rotate shoulders through the ball
	14	Decelerate club
	15	Direct clubhead to planned position
	16	Look to the outcome

*Note: Each movement phase contains basic action concepts that are functionally related to one another*.

To measure mental representation structure, a splitting task was conducted (step 1 of the SDA-M). It aimed to acquire the data on the representational distance among the 16 cognitive units (16 BACs) for golf putting. Participants were instructed to judge whether an anchor concept and a concept were functionally related to each other during the execution of the movement. Each of 16 BACs was presented as an anchor concept, whereas the remaining 15 BACs were provided, one by one in a random order, to be compared with the anchor concept. The anchor concept was not changed to a different concept until it was compared to each of the remaining 15 BACs. Once one process for an anchor concept was finished, another BAC was changed to the role as an anchor. This procedure was performed for each of 16 BACs (for more details see Schack, 2012).

#### Skill Performance

All participants were asked to perform a golf putt toward a golf hole (10.8 cm in diameter) projected by a beam at a distance of 3 m from the starting point on a synthetic putting green (length = 9 m, width = 4 m). Specifically, participants were required to putt a golf ball as close as possible to the target. To assess the accuracy of the performance, the two-dimensional position coordinate, of where the ball stopped after each golf putt trial, was recorded with six T10 CCD cameras (Vicon Motion Systems Ltd., Oxford, UK). Based on the collected data, the mean radial error (MRE) was calculated, which reflected the accuracy of the performance (e.g., Hancock et al., 1995).

#### Imagery Ability

The revised version of the Movement Imagery Questionnaire (MIQ-R; Hall and Martin, 1997) was used for the measurement of visual and kinesthetic imagery ability. The MIQ-R was composed of eight items: four items for visual imagery and four items for kinesthetic imagery. Participants were asked to execute a movement that was specified in each item. Then, participants were instructed to imagine the same movement that they had executed either visually or kinesthetically, without performing any actual movement. Next, they were required to rate how easy or difficult it was to imagine the movement on a seven-point Likert scale, ranging from 1 (very difficult to see or feel) to 7 (very easy to see or feel). The MIQ-R was found to have adequate internal reliability coefficients (0.84 for visual imagery subscales and 0.88 for kinesthetic imagery subscales) and sufficient test-retest reliability coefficients (0.80 for visual imagery subscales and 0.88 for kinesthetic imagery subscales; Monsma et al., 2009).

#### Post-Experimental Questionnaire

The participants of the action observation training and motor imagery training groups were asked to complete a post-experimental questionnaire immediately after the practice session of each day, which aimed to examine how easily they observed or imagined a movement. More specifically, the participants of the observation training group were required to indicate, on a seven-point Likert scale, anchored by 1 = “very difficult” to 7 = “very easy”, how easy it was to observe in accordance with the instruction. The participants of the motor imagery training group were also asked to rate based on a seven-point Likert scale. The scale ranged from “very difficult” at 1 to “very easy” at 7, which indicated how easy or difficult it was to imagine the movement according to the instruction.

### Procedure

This study was composed of a pre-test, 3 days of an experimental treatment, a post-test, and a retention test (see Table 2).

**Table 2:** Experimental procedure by group and time period.

	Pre-test	Treatment	Post-test	Retention test
Group	Day 1	Day 1 Day 2 Day 3	Day 3	Day 5
Action observation training group (*n* = 10)	SDA-M + Putting task	Observation training 60 times per day	SDA-M + Putting task	SDA-M + Putting task
Motor imagery training group (*n* = 10)	SDA-M + Putting task	Imagery training 60 times per day	SDA-M + Putting task	SDA-M + Putting task
Physical practice group (*n* = 10)	SDA-M + Putting task	Physical practice 60 times per day	SDA-M + Putting task	SDA-M + Putting task
No practice group (*n* = 10)	SDA-M + Putting task	No practice	SDA-M + Putting task	SDA-M + Putting task

*Note: SDA-M (structural dimensional analysis of mental representation): the psychometric method for measuring the mental representation structure of golf putting; putting task: 15 putting trials after two warm-up trials*.

#### Pre-Test

All participants participated individually in this experiment. A pre-test was carried out before beginning the experiment to evaluate the initial state of the mental representation structure and putting performance. Specifically, participants were asked to read and sign an informed consent form. Then, they were provided with an explanation regarding the splitting task and the meaning of each of 16 BACs of the putt. The splitting task was conducted to measure mental representation structure. After that, participants were required to perform two practice trials of golf putting followed by 15 test trials and try to putt the ball as close as possible to the target. After testing, participants were asked to complete the MIQ-R (Hall and Martin, 1997).

#### Experimental Treatment

Participants in experimental groups took part in a training program for 3 days, and performed 60 trials per day. In contrast, participants in the control group did not receive any training.

##### Action observation training group

Participants in the action observation training group performed 60 observational trials on each day of the practice phase. More specifically, they were requested to observe a video of a putting scene that showed putts completed by an expert golfer. The video was displayed with a first-person viewpoint of the expert model. A screen (1.5 × 1.5 m) was located in front of each participant. Participants were instructed to make a putting posture, and grasp the putter on the green as if they were actually performing the putting. Then, they were required to observe the putting scene as attentively as possible without actually performing it. There was a small break each time 20 observational trials were completed. The observational training was followed by the completion of a post-experimental questionnaire on how easily they observed the movement.

##### Motor imagery training group

Participants in the motor imagery training group conducted 60 mental trials on each day of the practice phase. Specifically, they were asked to imagine the full putting scene from the starting position on the green until the ball stopped on the target, without actually performing the putt, however. Their main task was to imagine the putting scene as clearly and vividly as possible, and for it to feel as real as possible, from their internal perspective. Participants were instructed to make a putting posture by grasping the putter on the green as if they were actually performing the putting. Then, they were required to imagine, with their eyes closed and at their own pace, that they raised one of their five fingers each time they completed the imagery of one putt. There was a small break after each time the imagery was performed 20 times. Following the mental training, participates completed a post-experimental questionnaire regarding how easily they imagined the movement.

##### Physical practice group

Participants in the physical practice group performed 60 physical trials for the putt on each day of the practice phase. Specifically, they were requested to putt the ball toward the positioned target that was 3 m away from starting point, in such a way that the ball rolled and then stopped as close to the target as possible. The putting practice was conducted at their own pace, and there was a short break each time the putting was performed 20 times. No feedback was provided, except for the immediate visible outcome of each putt.

##### Control group

Participants assigned to the control group did not participate in any training for 3 days of training period.

#### Post-Test and Retention Test

The post- and retention tests were administered the day after the end of the experiment, and 2 days after the post-test, respectively. The experimental protocol and the items measured were identical to those from the pre-test except for completing the MIQ-R in pre-test.

### Data Analysis

#### Imagery Ability

A one-way ANOVA was used independently for the kinesthetic imagery score, visual imagery score, and the combined score to examine whether there was a difference in imagery ability for basic body movements among the four groups.

#### Post-Experimental Questionnaire

ANOVA was used to compare the action observation training and the motor imagery training groups’ results of the post-experimental questionnaire, which was performed during the 3 days of each treatment. Two-way ANVOAs (two cognitive training groups × three practice days) with repeated measures on the last factor were conducted to compare the difference between the ease of action observation and motor imagery. The significance level for data analysis was set at 5%.

#### Mental Representation Structure

Cluster analysis was conducted based on the data extracted from the splitting task, which aimed to assess not only how many significant clusters there were in the mean dendrogram by group and test session, but also how well-structured their relations were (e.g., Schack, 2012). The significance level for cluster analysis was set at 5%, which corresponded to a critical value of 3.41 (i.e., *d*_crit_ = 3.41). The critical value (*d*_crit_) was displayed as the horizontal line on the dendrogram. The clusters below the line were considered statistically significant. Then, statistical analysis of invariance was performed to examine statistical differences in mental representation structure according to group and test session (e.g., Schack, 2012; Frank et al., 2013). For invariance analysis, a critical value was *λ* = 0.68. A value equal to or more than 0.68 (i.e., *λ* ≥ 0.68) indicated that there was no difference (two cluster solutions were invariant), while a value less than 0.68 (i.e., *λ* < 0.68) indicated that there was change (two cluster solutions were variant). In addition, the Adjusted Rand Index (ARI; e.g., Santos and Embrechts, 2009) was calculated to evaluate the degree of similarity between a group dendrogram and a reference dendrogram, reflecting well the four phases of the golf putt (e.g., Frank et al., 2013). The value of ARI ranged from “−1” to “1”. The value “−1” or “1” indicated “completely different” or “completely same” in terms of the degree of similarity, respectively.

#### Skill Performance

MRE was calculated based on two-dimension coordinates of each putting trial, which reflected the accuracy of putting performance (e.g., Hancock et al., 1995). The calculated variable was analyzed by two-way ANOVAs (four groups × three test sessions) with the repeated measures on the last factor. In addition, further analysis was conducted with a one-way ANOVA to investigate differences between test sessions by group, and vice versa. The significance level for all analyses was set at 5%.

#### Correlation

A two-tailed Pearson’s correlation coefficient analysis was performed to investigate the relationship between the change of mental representation structure over time (i.e., from pre-, to post-, and to retention test) and the change of skill performance over time. Adjusted rand index, which reflects the similarity between each individual’s mental structure and the expert reference mental structure, and mean radial error, which reflects the accuracy of skill performance, were used for the analysis of the correlation. The significance level for all analyses was set at 5%.

## Results

### Imagery Ability

The assessment result for general imagery ability showed that there was no main effect of group for the visual imagery score, *F*_(3,36)_ = 0.389, *p* = 0.762, $\eta_{\text{p}}^{\text{2}}$ = 0.031, the kinesthetic imagery score, *F*_(3,36)_ = 0.366, *p* = 0.778, $\eta_{\text{p}}^{\text{2}}$ = 0.030, and the combined score, *F*_(3,36)_ = 0.157, *p* = 0.925, $\eta_{\text{p}}^{\text{2}}$ = 0.013. This meant that there was no difference among groups in imagery ability before the experiment began. Furthermore, the mean score for the three imagery variables was 5.80, 5.33 and 5.40, respectively. Specifically, each group scored an average of five points (i.e., somewhat easy to see or feel) or more, for visual, kinesthetic, and overall imagery scores. This indicated that each group had adequate imagery ability (e.g., Smith and Collins, 2004; Smith et al., 2008; Frank et al., 2014).

### Post-Experimental Questionnaire

The analysis result for the ease of complying with the instruction showed that the main effect of group, *F*_(1,18)_ = 4.987, *p* = 0.038, $\eta_{\text{p}}^{\text{2}}$ = 0.217 and practice day, *F*_(2,36)_ = 17.934, *p* = 0.000, $\eta_{\text{p}}^{\text{2}}$ = 0.499, was significant, respectively. However, it was revealed that a significant interaction between group and practice day, *F*_(2,36)_ = 4.796, *p* = 0.014, $\eta_{\text{p}}^{\text{2}}$ = 0.210, was also significant. For the *post hoc* test, the difference between groups by practice day (or vice versa) was analyzed. The result of the first *post hoc* test showed that on the first and second practice day, the score of the action observation training group was significantly higher than the score of the motor imagery training group for the ease of complying with the instruction (*p* = 0.020, *p* = 0.017). However, it was revealed that there was no significant difference between the two groups on the last practice day (*p* = 0.458). In addition, the result of the second *post hoc* test showed that for the action observation training group, the score of the second practice day had significantly increased compared to the score of the first practice day (*p* = 0.045), and such an increased score was maintained on the last practice day (*p* = 0.157). In contrast, for the motor imagery training group, it was shown that there was no significant difference between the first and second practice day (*p* = 0.153), whereas the score of the third practice day was higher than the score of the first (*p* = 0.002) and second practice day (*p* = 0.032).

### Mental Representation Structure

#### Action Observation Training Group

**Figure 1:**
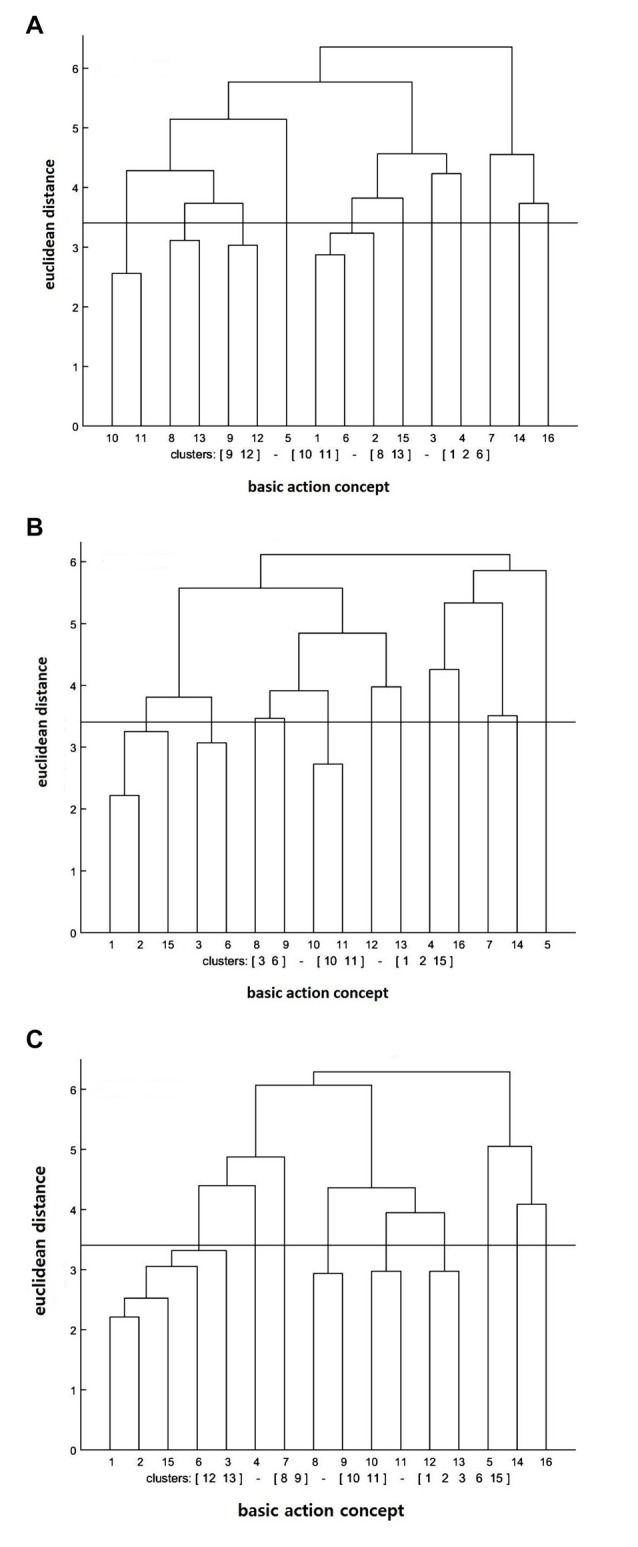
Mean dendrograms indicating mental representation structure of action observation training group at **(A)** pre-test, **(B)** post-test and **(C)** retention test. The horizontal line indicates the critical Euclidean distance. The critical value of the Euclidean distance (*d_crit_*_)_ was 3.41 for an α level of 5%. The basic action concepts (BACs) above this line are considered not related. The underlined BACs below this line are considered functionally related to each other.

The cluster analysis showed that the number of statistically significant functional clusters had increased over the test sessions (see Figure 1). More specifically, the clusters were (BAC 1, 2, 6), (BAC 8, 13), (BAC 9, 12), (BAC 10, 11) at the pre-test, (BAC 1, 2, 15), (BAC 3, 6), (BAC 10, 11) at the post-test, and (BAC 1, 2, 3, 6, 15), (BAC 8, 9), (BAC 10, 11), (BAC 12, 13) at the retention test. Furthermore, the invariance analysis was conducted to determine whether there was a statistically significant difference between the test sessions. The invariance analysis indicated that there was an evident significant difference between the pre- and post-tests (*λ* = 0.37), between the post- and retention tests (*λ* = 0.42), and between the pre- and retention tests (*λ* = 0.48). Lastly, to evaluate the degree of similarity between the mean dendrogram of the action observation training group and the reference dendrogram that was composed of four phases (i.e., preparation BACs 1–4, backswing BACs 5–7, forward swing BACs 8–11, and attenuation BACs 12–16), the ARI was calculated. The ARI analysis revealed that the similarity became higher over the test session, given that the ARI value ranges from −1 (i.e., completely different) to +1 (i.e., completely same). Specifically, ARI_pre_ = 0.05, ARI_post_ = 0.07, and ARI_retention_ = 0.10 were shown at pre-, post-, and retention tests, respectively.

#### Motor Imagery Training Group

**Figure 2:**
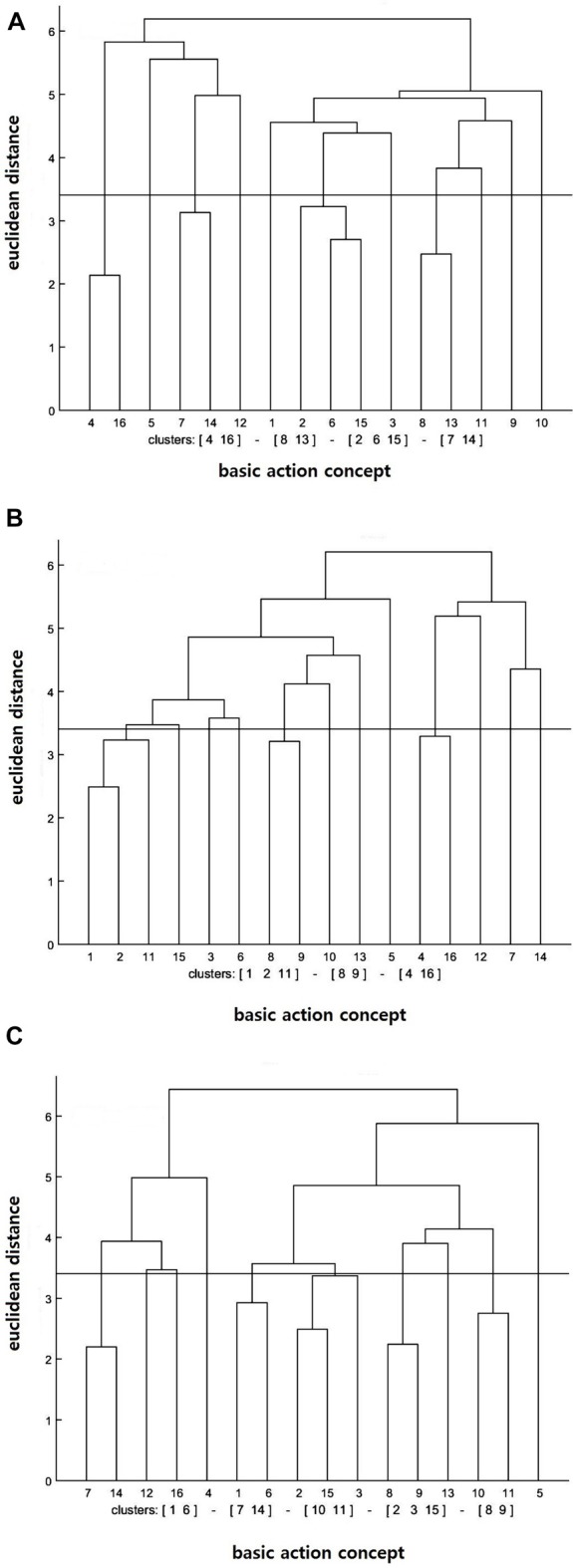
Mean dendrograms indicating mental representation structure of motor imagery training group at **(A)** pre-test, **(B)** post-test and **(C)** retention test (*α* = 0.05; *d*_crit_ = 3.41).

Similar to the result of the action observation training group, the cluster analysis of the motor imagery training group demonstrated that the number of statistically significant functional clusters had increased over the test sessions (see Figure 2). More particularly, the clusters were (BAC 2, 6, 15), (BAC 4, 16), (BAC 7, 14), (BAC 8, 13) at the pre-test, (BAC 1, 2, 11), (BAC 4, 16), (BAC 8, 9) at the post-test, and (BAC 1, 6), (BAC 2, 3, 15), (BAC 7, 14), (BAC 8, 9), (BAC 10, 11) at the retention test. In addition, the invariance analysis showed that there was a significant difference between the pre- and post-tests (*λ* = 0.33), between the post- and retention tests (*λ* = 0.33), and between the pre- and retention tests (*λ* = 0.30). Lastly, the ARI analysis showed that the similarity between the mean dendrogram of the motor imagery training group and the reference dendrogram increased over the test sessions. Precisely, ARI_pre_ = 0.01, ARI_post_ = 0.02, and ARI_retention_ = 0.04 were displayed at the pre-, post-, and retention tests, respectively.

#### Physical Practice Group

**Figure 3:**
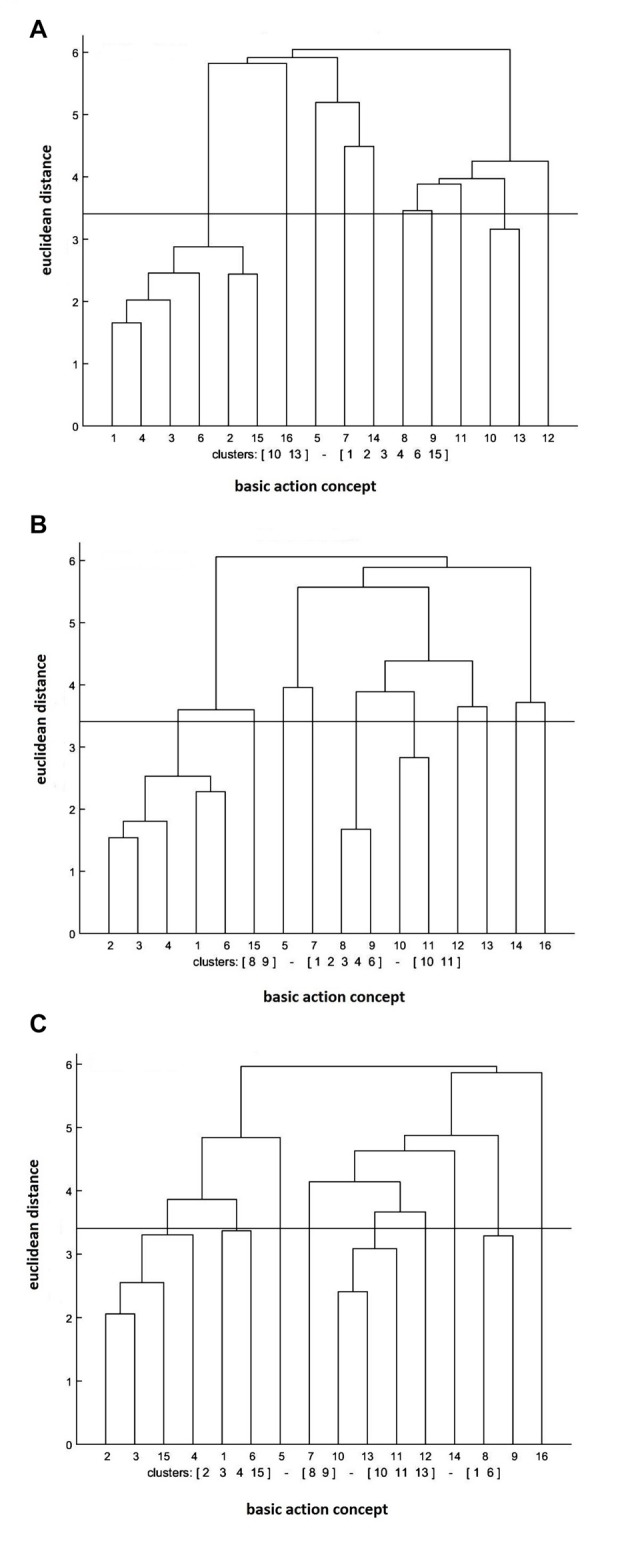
Mean dendrograms indicating mental representation structure of physical practice group at **(A)** pre-test, **(B)** post-test and **(C)** retention test (*α* = 0.05; *d*_crit_ = 3.41).

The cluster analysis of the physical practice group revealed that the increase in the number of significant functional clusters was evident over the test session (see Figure 3). In further detail, the clusters were (BAC 1, 2, 3, 4, 6, 15), (BAC 10, 13) at the pre-test, (BAC 1, 2, 3, 4, 6), (BAC 8, 9), (BAC 10, 11) at the post-test, and (BAC 1, 6), (BAC 2, 3, 4, 15), (BAC 8, 9), (BAC 10, 11, 13) at the retention test. Moreover, the invariance analysis demonstrated that there was a significant difference between the pre- and post-tests (*λ* = 0.45), between the post- and retention tests (*λ* = 0.45), and between the pre- and retention tests (*λ* = 0.40). Lastly, the ARI analysis showed that the mean dendrogram of the physical practice group and the reference dendrogram became more similar over the test sessions. Specifically, ARI_pre_ = 0.08, ARI_post_ = 0.11, and ARI_retention_ = 0.13 were shown at the pre-, post-, and retention tests, respectively.

#### Control Group

**Figure 4:**
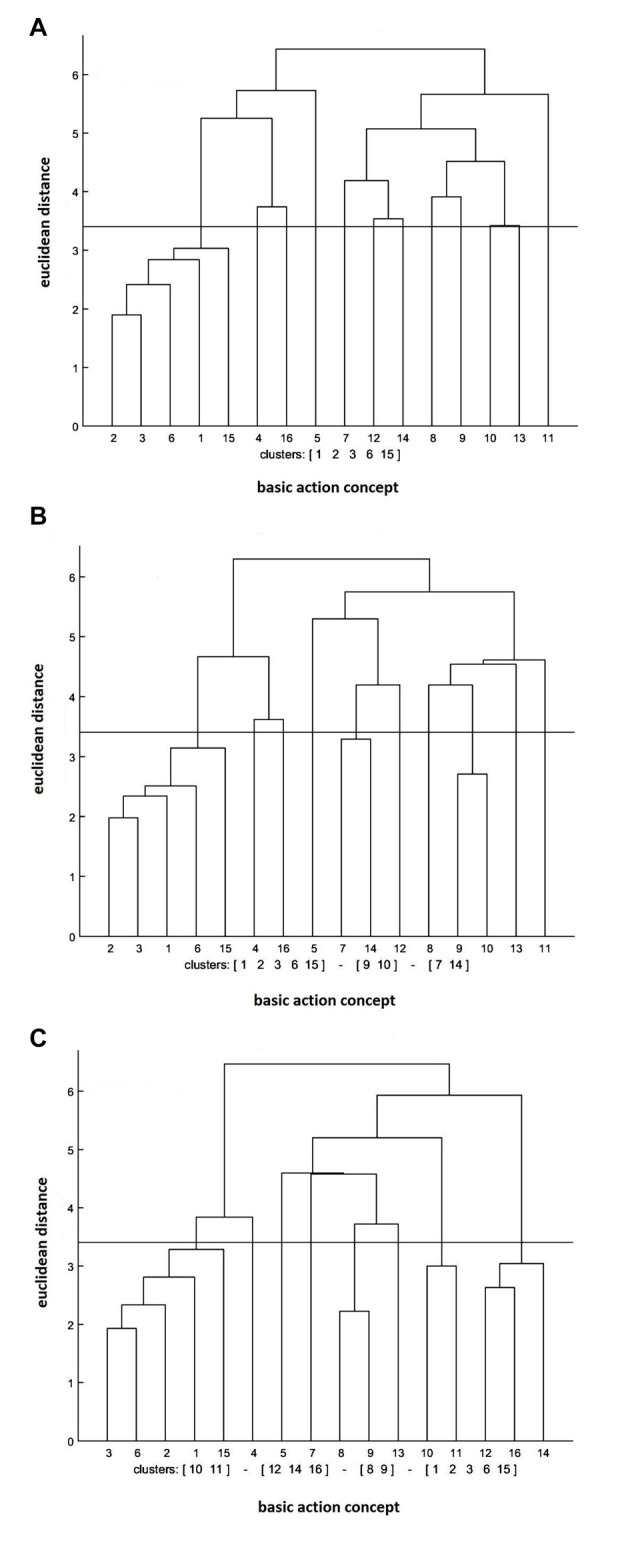
Mean dendrograms indicating mental representation structure of control group at **(A)** pre-test, **(B)** post-test and **(C)** retention test (*α* = 0.05; *d*_crit_ = 3.41).

The cluster analysis of the control group revealed that the number of functional clusters had increased significantly over the test sessions (see Figure 4). More specifically, the clusters were (BAC 1, 2, 3, 6, 15) at the pre-test, (BAC 1, 2, 3, 6, 15), (BAC 7, 14), (BAC 9, 10) at the post-test, and (BAC 1, 2, 3, 6, 15), (BAC 8, 9), (BAC 10, 11), (BAC 12, 14, 16) at the retention test. Furthermore, the invariance analysis revealed that there was a significant difference between the pre- and post-tests (*λ* = 0.38), between the post- and retention tests (*λ* = 0.45), and between the pre- and retention tests (*λ* = 0.42). Lastly, the ARI analysis demonstrated that the mean dendrogram of the control group became more similar to the reference dendrogram over the test sessions. To be exact, ARI_pre_ = 0.02, ARI_post_ = 0.03, and ARI_retention_ = 0.07 were revealed for the pre-, post-, and retention tests, respectively.

### Accuracy of Performance

**Figure 5:**
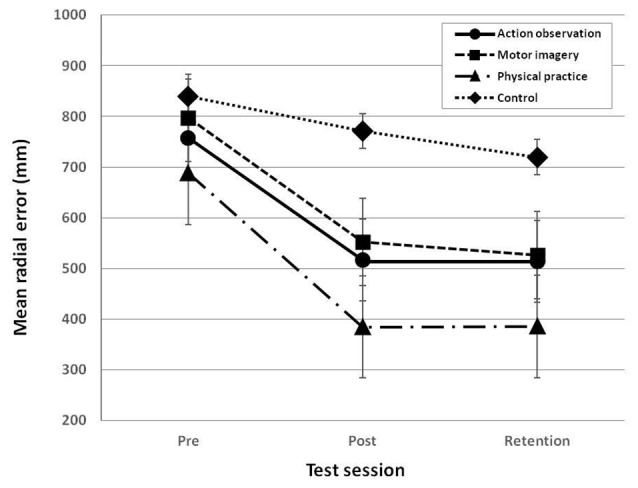
Mean radial error (MRE) across group and test session. MRE reflects the accuracy of the putting performance. Error bars indicate standard errors.

The analysis of the accuracy of putting performance revealed that the main effects of group, *F*_(3,36)_ = 4.583, *p* = 0.008, $\eta_{\text{p}}^{\text{2}}$ = 0.276, and test session, *F*_(2,72)_ = 24.744, *p* = 0.000, $\eta_{\text{p}}^{\text{2}}$ = 0.407, were significant, respectively (see Figure 5). The result of the *post hoc* test on the main effect of group showed that the physical practice group only performed significantly better than the control group, *p* = 0.005, *d* = 1.25, and that no significant differences were found among the AO training group, the MI training group, and the control group. In addition, the result of the *post hoc* test on the main effect of test session showed that the performance of the post-test, *p* = 0.000, *d* = 1.03 and the retention test, *p* = 0.000, *d* = 1.06, was significantly better than the performance of the pre-test, respectively, and that no significant difference was found between the post-test and the retention test. Moreover, the result of the *post hoc* test on the differences among test sessions by group showed that the accuracy in the post-test of the AO training group, *p* = 0.037, *d* = 0.96, the MI training group, *p* = 0.006, *d* = 1.50, and the PP group, *p* = 0.005, *d* = 1.55, was significantly higher than the accuracy of the pre-test, respectively, and that such an improvement was maintained in the retention test. However, the control group did not show significant differences among the pre-test, the post-test, and the retention test, *p* = 0.266, $\eta_{\text{p}}^{\text{2}}$ = 0.137. In addition, the result of the *post hoc* test on the differences groups by test session showed that the accuracy of the AO training group, *p* = 0.034, *d* = 0.97, and the PP group, *p* = 0.000, *d* = 1.56, was significantly higher than the control group in the post-test, and that the accuracy of the PP group, *p* = 0.009, *d* = 1.26, was significantly better than the control group in the retention test. For the pre-test, no significant differences in the accuracy were found among groups, *p* = 0.587, $\eta_{\text{p}}^{\text{2}}$ = 0.052.

### Correlation

**Figure 6:**
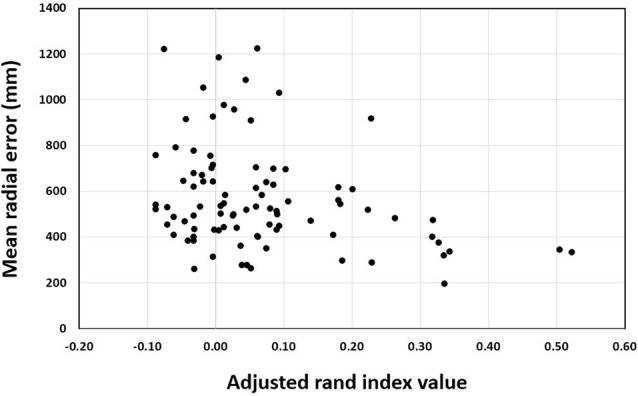
Pearson correlation between adjusted rand index and mean radial error over test sessions across all training groups.

**Figure 7:**
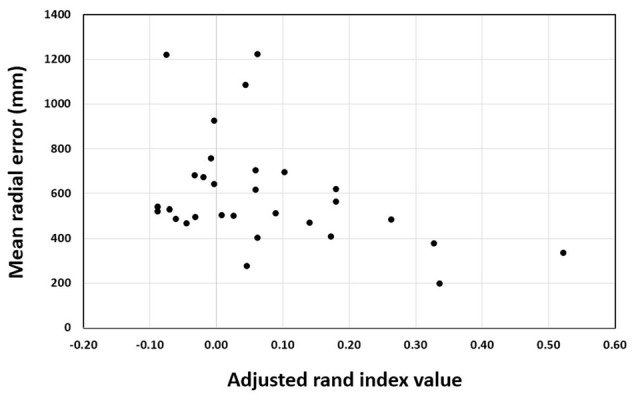
Pearson correlation between adjusted rand index and mean radial error over test sessions of the action observation training group.

For the training groups (i.e., action observation training, motor imagery training and physical practice), the Pearson correlation between ARI and MRE was significant, *r* = −0.307, *n* = 90, *p* = 0.003 (see Figure 6). This indicates that the more elaborate the representation as shown by higher rand indices, the better the performance was as indicated by lower error scores. In addition, the correlation analysis for the action observation training group revealed that the Pearson correlation between ARI and MRE was significant, *r* = −0.399, *n* = 30, *p* = 0.029 (see Figure 7). This also indicates that the relationship between mental representation structure and skill performance is positive for the action observation training group. However, for the motor imagery training group, *r* = 0.010, *n* = 30, *p* = 0.956, the physical practice group, *r* = −0.241, *n* = 30, *p* = 0.200, and the control group, *r* = 0.135, *n* = 30, *p* = 0.477, the Pearson correlation between the ARI and MRE was not significant.

## Discussion

The purpose of the present study was to investigate the influence of action observation training and motor imagery training on the development of mental representation, as well as the performance of motor skill. Specifically, this study was designed to compare the effects of the two cognitive training interventions for both mental representation structure and skill performance, as well as to examine the relationship between the change of mental representation structure and the change of skill performance. In this respect, the present study is an extension of previous research done by Kim et al. (2011), which have studied the effect of action observation training and motor imagery training on skill performance. It was hypothesized that action observation training would be more effective than motor imagery training, both in the development of mental representation structure and in the improvement of skill performance in early stage of skill learning. Furthermore, it was expected that the change in mental representation structure would be connected with the change in skill performance. The results of this study support the established hypothesis partially. To be specific, we could find some meaningful improvement in mental representation structure and skill performance over time through both action observation training and motor imagery training. In addition, it was found that changes in mental representation structure and skill performance were positively correlated for the action observation training group.

With regard to mental representation structure of golf putting, it was revealed that the mental representation structures of all practice groups (i.e., motor imagery, action observation and physical practice) changed over time, leading to more elaborate and structured representations in the direction of the expert reference dendrogram, reflecting well the four movement phases (i.e., BAC 1–4, BAC 5–7, BAC 8–11 and BAC 12–16; Frank et al., 2013). This result indicates that practice brings about functional changes of task-specific mental representation in long-term memory, which is in line with results of recent studies on perceptual-cognitive changes (Frank et al., 2013, 2014, 2016).

Related to this, studies focusing on the neurophysiological mechanisms underlying motor imagery and action observation have shown that the two simulation states of action share certain neural representations (Conson et al., 2009; Holmes et al., 2010). For instance, brain regions such as the primary and the premotor cortex, the supplementary motor area, the cerebellum and the basal ganglia tend to be activated not only during motor imagery, but also during action observation in the absence of any overt action (Lorey et al., 2013). In this respect, the changes in mental representation structure in this study may be associated with changes in brain activation that may in turn be correlated with motor outcome. The extent to which each simulation state contributes to changes in motor learning related brain areas, however, has yet to be determined (Frank and Schack, 2017).

Interestingly, it was revealed that the initial status of the mental representation structure differed somewhat between the groups. Specifically, (ARI_pre_ = 0.05, BAC 1, 2, 6; BAC 8, 13; BAC 9, 12; BAC 10, 11), (ARI_pre_ = 0.01, BAC 2, 6, 15; BAC 4, 16; BAC 7, 14; BAC 8, 13), (ARI_pre_ = 0.08, BAC 1, 2, 3, 4, 6, 15; BAC 10, 13), and (ARI_pre_ = 0.02, BAC 1, 2, 3, 6, 15) meaningful clusters (of golf putting) were shown for the pre-test in action observation training, motor imagery training, physical practice, and control groups, in that order. Meaningful clusters represent a group of BACs that are functionally or biomechanically related to movement components and phases for the achievement of action goals (Schack, 2012). Thus, this result indicates that even though participants actually had no previous performance experience with the task, the initial cognitive architecture of the mental representation may have varied depending upon the individual, suggesting that the initial dissimilarity of mental representation structure may lead to different effects for the development of mental representation and performance during the early motor learning phase. However, as Frank et al. (2014) indicated in their article, to date, it is not clear whether the slope of representation development is functionally related to the initial status of representation structure. The rate of change in representation is likely to be different because there are various representation structures in novices. Therefore, future studies should be undertaken to investigate differences in the development of mental representation and skill performance according to the degree of novices’ initial representation structure for a better understating of this issue.

Contrary to our expectation, the mental representation structure of the control group developed over time as well. Specifically, (BAC 1, 2, 3, 6, 15) at the pre-test (ARI_pre_ = 0.02), (BAC 1, 2, 3, 6, 15), (BAC 7, 14), (BAC 9, 10) at the post-test (ARI_post_ = 0.03), and (BAC 1, 2, 3, 6, 15), (BAC 8, 9), (BAC 10, 11), (BAC 12, 14, 16) at the retention test (ARI_retention_ = 0.07) were found respectively. The possible explanation for this result is that physical test trials executed during test sessions might have influenced the development of the mental representation structure.

With reference to the cognitive effect of the action observation training and motor imagery training, it was expected that action observation training, associated with a bottom-up mechanism, would be more effective than motor imagery training, associated with a top-down mechanism, in the development of a mental representation structure for novices in the early learning stage (see Holmes and Calmels, 2008 for details on potential mechanisms involved in observation and imagery). However, we could not compare the difference in mental representation structure objectively between the two cognitive trainings, due to the difference between the initial representation structures. Nevertheless, the result of this study is meaningful in that task-specific representation structure can be developed through practice, which is especially relevant to the perceptual-cognitive perspective (Mechsner et al., 2001), and the cognitive action architecture approach (Schack, 2004), which emphasize the crucial role of mental representation for the generation and control of voluntary movements.

Regarding the ease of the use of the two types of cognitive trainings, the present result of the post-experimental questionnaire showed that the use score of action observation was significantly higher than that of motor imagery by the second practice day, out of the 3 days in the practice period. This result indicates that action observation was easier to use than motor imagery for novices who had no previous task experience. For example, Soohoo et al. (2001) compared the effect of observation and imagery on the performance of a squat lift skill. In their study, participants were instructed to choose and use their preferred method of the two types of cognitive training (i.e., action observation and motor imagery) for the fifth trial, after they performed the fourth trial of either observation or imagery in each predetermined group. The result of the study demonstrated that many participants preferred to watch the video of an expert model who performed the task. This result shows that novices prefer action observation to motor imagery during the early stage of skill learning. However, there are still individual differences associated with preference. Therefore, in future research, individual preference needs to be considered for the assignment of cognitive training groups.

With regard to performance outcome, first, before the practice phase, there was no difference in the accuracy among the four groups. After 3 days of training, the three practice groups (i.e., action observation training, motor imagery training, and physical practice group), except for the control group, significantly improved in accuracy at the post-test compared to the pre-test. Moreover, such an improvement was maintained at the retention test. This is in line with the research result of Kim et al. (2011) that investigated the difference in learning effect between action observation and motor imagery of golf putting. Thus, this result pattern supports the previous findings that not only physical practice, but also cognitive training such as action observation training or motor imagery training, can enhance motor skill acquisition and learning (Driskell et al., 1994; Wulf et al., 2005; Hayes et al., 2007; Schmidt and Lee, 2013). In addition, as Frank et al. (2014) mentioned in their article, which investigated functional links between motor memory and motor imagery, the relative short length of practice may be one reason for the lack of differences among the groups in the performance of golf putting. Therefore, future research on this topic that employs golf putt task needs to consider a longer practice period to prevent possible confounds in statistic result.

Lastly, regarding the relationship between the change of mental representation structure and the change of skill performance, it was revealed that the change in mental representation structure of the training groups was positively related to the change in skill performance, with more elaborate mental representation being linked to better skill performance. This result indirectly supports the perceptual-cognitive perspective, which emphasizes the role of mental representation as a basis for the control of voluntary motor movements (Mechsner et al., 2001; Schack and Mechsner, 2006). Additionally, the positive relationship between the two variables by group was found to be significant for only action observation group. This result implies that the mental representation elaborated through action observation training might be more related to the execution of the motor system.

Taken together, the findings of this study provide insights into perceptual-cognitive and performance changes in the process of motor learning through cognitive training. This study is the first to compare the effect of action observation training and motor imagery training on both the development of mental representation and skill performance. Moreover, it is noteworthy that this study has demonstrated that the two simulation states of action led to both cognitive adaptation and skill improvement, and that action observation training resulted in the positive relationship between cognitive-perceptual and performance changes. However, the generalizability of these findings is subject to certain limitations. For instance, although this study was designed to compare action observation and motor imagery as objectively as possible, it was difficult to control all of the factors that can influence the effect of action observation and motor imagery, such as the possibility of imagery during observation or the initial representation status of participants. Thus, more research is required to confirm the findings of the present study. Regarding future research on action observation and motor imagery, future research might investigate the perceptual-cognitive and behavioral patterns of the combined training (i.e., AO + MI training) over the course of learning or relearning in motor skill learning and motor rehabilitation settings rather than their independent use (Eaves et al., 2016).

## Author Contributions

TK, CF and TS: conception and design of study and drafting of manuscript; TK and CF: experiment preparation and data analysis; TK: data collection.

## Conflict of Interest Statement

The authors declare that the research was conducted in the absence of any commercial or financial relationships that could be construed as a potential conflict of interest.

## References

[B1] Bajaj S. Butler A. J. Drake D. Dhamala M. (2015). Functional organization and restoration of the brain motor-execution network after stroke and rehabilitation. Front. Hum. Neurosci. 9:173. 10.3389/fnhum.2015.00173 25870557 PMC4378298

[B2] Bandura A. (1986). Social Foundations of Thought and Action: A Social-Cognitive View. Englewood Cliffs, NJ: Prentice-Hall.

[B3] Bandura A. (1997). Self-Efficacy: The Exercise of Control. New York, NY: Freeman.

[B5] Bläsing B. Tenenbaum G. Schack T. (2009). The cognitive structure of movements in classical dance. Psychol. Sports Exerc. 10, 350–360. 10.1016/j.psychsport.2008.10.001

[B6] Clark S. Tremblay F. Ste-Marie D. (2004). Differential modulation of corticospinal excitability during observation, mental imagery and imitation of hand actions. Neuropsychologia 42, 105–112. 10.1016/s0028-3932(03)00144-1 14615080

[B7] Coker C. A. (2004). Motor Learning and Control for Practitioners. Boston, MA: McGraw-Hill Humanities/Social Sciences/Languages.

[B8] Conson M. Sarà M. Pistoia F. Trojano L. (2009). Action observation improves motor imagery: specific interactions between simulative processes. Exp. Brain Res. 199, 71–81. 10.1007/s00221-009-1974-3 19690843

[B9] Decety J. (1996). The neurophysiological basis of motor imagery. Behav. Brain Res. 77, 45–52. 10.1016/0166-4328(95)00225-1 8762158

[B10] Driskell J. E. Copper C. Moran A. (1994). Does mental practice enhance performance? J. Appl. Psychol. 79, 481–492. 10.1037/0021-9010.79.4.481

[B11] Eaves D. Riach M. Holmes P. Wright D. (2016). Motor imagery during action observation: a brief review of evidence, theory and future research opportunities. Front. Neurosci. 10:514. 10.3389/fnhum.2017.00025 27917103 PMC5116576

[B12] Farah M. J. (1984). The neurological basis of mental imagery: a componential analysis. Cognition 18, 245–272. 10.1016/0010-0277(84)90026-x 6396031

[B13] Filimon F. Nelson J. D. Hagler D. J. Sereno M. I. (2007). Human cortical representations for reaching: mirror neurons for execution, observation, and imagery. Neuroimage 37, 1315–1328. 10.1016/j.neuroimage.2007.06.008 17689268 PMC2045689

[B16] Frank C. Land W. M. Popp C. Schack T. (2014). Mental representation and mental practice: experimental investigation on the functional links between motor memory and motor imagery. PLoS One 9:e95175. 10.1371/journal.pone.0095175 24743576 PMC3990621

[B14] Frank C. Land W. M. Schack T. (2013). Mental representation and learning: the influence of practice on the development of mental representation structure in complex action. Psychol. Sports Exerc. 14, 353–361. 10.1016/j.psychsport.2012.12.001

[B15] Frank C. Land W. M. Schack T. (2016). Perceptual-cognitive changes during motor learning: the influence of mental and physical practice on mental representation, gaze behavior, and performance of a complex action. Front. Psychol. 6:1981. 10.3389/fpsyg.2015.01981 26779089 PMC4705276

[B90] Frank C. Schack T. (2017). The representation of motor (inter)action, states of action, and learning: three perspectives on motor learning by way of imagery and execution. Front. Psychol. 8:678. 10.3389/fpsyg.2017.00678 28588510 PMC5440750

[B18] Guillot A. Collet C. (2010). The Neurophysiological Foundations of Mental and Motor Imagery. New York, NY: Oxford University Press.

[B20] Hall C. R. Buckolz E. Fishburne G. J. (1992). Imagery and the acquisition of motor skills. Can. J. Sports Sci. 17, 19–27. 1322764

[B19] Hall C. R. Martin K. A. (1997). Measuring movement imagery abilities: a revision of the movement imagery questionnaire. J. Ment. Imagery. 21, 143–154.

[B21] Hancock G. R. Butler M. S. Fischman M. G. (1995). On the problem of two-dimensional error scores: measures and analyses of accuracy, bias, and consistency. J. Mot. Behav. 27, 241–250. 10.1080/00222895.1995.9941714 12529235

[B22] Hayes S. J. Ashford D. Bennett S. J. (2008). Goal-directed imitation: the means to an end. Acta Psychol. 127, 407–415. 10.1016/j.actpsy.2007.07.009 17880901

[B23] Hayes S. J. Hodges N. J. Huys R. Mark Williams A. (2007). End-point focus manipulations to determine what information is used during observational learning. Acta Psychol. 126, 120–137. 10.1016/j.actpsy.2006.11.003 17204236

[B24] Helm F. Marinovic W. Krüger B. Munzert J. Riek S. (2015). Corticospinal excitability during imagined and observed dynamic force production tasks: effortfulness matters. Neuroscience 290, 398–405. 10.1016/j.neuroscience.2015.01.050 25639231

[B25] Hodges N. J. Williams A. M. (Eds). (2012). Skill Acquisition in Sport: Research, Theory and Practice. 2nd Edn. London: Routledge.

[B26] Holmes P. Calmels C. (2008). A neuroscientific review of imagery and observation use in sport. J. Mot. Behav. 40, 433–445. 10.3200/JMBR.40.5.433-445 18782718

[B27] Holmes P. Cumming J. Edwards M. G. (2010). “Movement imagery, observation, and skill,” in The Neurophysiological Foundations of Mental and Motor Imagery, eds Guillot A. Collet C. (Oxford: Oxford University Press), 253–269.

[B28] Horn R. R. Williams A. M. Hayes S. J. Hodges N. J. Scott M. A. (2007). Demonstration as a rate enhancer to changes in coordination during early skill acquisition. J. Sports Sci. 25, 599–614. 10.1080/02640410600947165 17365545

[B30] Jeannerod M. (1995). Mental imagery in the motor context. Neuropsychologia 33, 1419–1432. 10.1016/0028-3932(95)00073-c 8584178

[B31] Jeannerod M. (2001). Neural simulation of action: a unifying mechanism for motor cognition. Neuroimage 14, S103–S109. 10.1006/nimg.2001.0832 11373140

[B91] Jeannerod M. Decety J. (1995). Mental motor imagery: a window into the representational stages of action. Curr. Opin. Neurobiol. 5, 727–732. 10.1016/0959-4388(95)80099-9 8805419

[B32] Kim T. Cruz A. Ha J. (2011). Differences in learning facilitatory effect of motor imagery and action observation of golf putting. J. Appl. Sci. 11, 151–156. 10.3923/jas.2011.151.156

[B33] Kohl R. M. Shea C. H. (1992). Observational learning: influences on temporal response organization. Hum. Perform. 5, 235–244. article. 10.1207/s15327043hup0503_4

[B34] Land W. M. Volchenkov D. Bläsing B. E. Schack T. (2013). From action representation to action execution: exploring the links between cognitive and biomechanical levels of motor control. Front. Comput. Neurosci. 7:127. 10.3389/fncom.2013.00127 24065915 PMC3776155

[B35] Liu H. Song L. P. Zhang T. (2014). Mental practice combined with physical practice to enhance hand recovery in stroke patients. Behav. Neurol. 2014:876416. 10.1155/2014/876416 25435713 PMC4241695

[B36] Lorey B. Naumann T. Pilgramm S. Petermann C. Bischoff M. Zentgraf K.. (2013). How equivalent are the action execution, imagery and observation of intransitive movements? Revisiting the concept of somatotopy during action simulation. Brain Cognit. 81, 139–150. 10.1016/j.bandc.2012.09.011 23207575

[B37] Lotze M. Montoya P. Erb M. Hülsmann E. Flor H. Klose U.. (1999). Activation of cortical and cerebellar motor areas during executed and imagined hand movements: an fMRI study. J. Cogn. Neurosci. 11, 491–501. 10.1162/089892999563553 10511638

[B38] Magill R. A. (2000). Motor Learning: Concepts and Applications. New York, NY: McGraw-Hill.

[B39] Mechsner F. Kerzel D. Knoblich G. Prinz W. (2001). Perceptual basis of bimanual coordination. Nature 414, 69–73. 10.1038/35102060 11689944

[B40] Monsma E. V. Short S. E. Hall C. R. Gregg M. Sullivan P. (2009). Psychometric properties of the revised movement imagery questionnaire (MIQ-R). J. Imagery Res. Sports Phys. Activ. 4:1 10.2202/1932-0191.1027

[B41] Mulder T. Zijlstra S. Zijlstra W. Hochstenbach J. (2004). The role of motor imagery in learning a totally novel movement. Exp. Brain Res. 154, 211–217. 10.1007/s00221-003-1647-6 14508635

[B42] Murphy S. M. (1994). Imagery interventions in sport. Med. Sci. Sports Exerc. 26, 486–494. 10.1249/00005768-199404000-00014 8201906

[B43] Ram N. Riggs S. M. Skaling S. Landers D. M. McCullagh P. (2007). A comparison of modelling and imagery in the acquisition and retention of motor skills. J. Sports Sci. 25, 587–597. 10.1080/02640410600947132 17365544

[B44] Santos J. M. Embrechts M. (2009). “On the use of the adjusted rand index as ametric for evaluating supervised classification,” in Artificial Neural Networks—ICANN, Lecture Notes in Computer Science, eds Alippi C. Polycarpou M. Panayiotou C. Ellinas G. (Berlin: Springer), 175–184.

[B45] Schack T. (2004). The cognitive architecture of complex movement. Int. J. Sports Exerc. Psychol. 2, 403–438. 10.1080/1612197x.2004.9671753

[B46] Schack T. (2012). “Measuring mental representations,” in Handbook of Measurement in Sport and Exercise Psychology, eds Tenenbaum I. G. Eklund R. C. Kamata A. (Champaign, IL: Human Kinetics), 203–214.

[B50] Schack T. Essig K. Frank C. Koester D. (2014). Mental representation and motor imagery training. Front. Hum. Neurosci. 8:328. 10.3389/fnhum.2014.00328 24904368 PMC4033090

[B47] Schack T. Hackfort D. (2007). “Action-theory approach to applied sport psychology,” in Handbook of Sport Psychology, eds Tenenbaum G. Ecklund R. C. (New Jersey, NJ: Wiley), 332–351.

[B48] Schack T. Mechsner F. (2006). Representation of motor skills in human long-term memory. Neurosci. Lett. 391, 77–81. 10.1016/j.neulet.2005.10.009 16266782

[B49] Schack T. Ritter H. (2009). The cognitive nature of action—functional links between cognitive psychology, movement science, and robotics. Prog. Brain Res. 174, 231–250. 10.1016/s0079-6123(09)01319-3 19477343

[B51] Schmidt R. Lee T. (2013). Motor Learning and Performance, 5E with Web Study Guide: From Principles to Application. Champaign, IL: Human Kinetics.

[B92] Shea C. H. Wright D. L. Wulf G. Whitacre C. (2000). Physical and observational practice afford unique learning opportunities. J. Mot. Behav. 32, 27–36. 10.1080/00222890009601357 11008269

[B52] Smith D. Collins D. (2004). Mental practice, motor performance and the late CNV. J. Sports Exerc. Pychol. 26, 412–426. 10.1123/jsep.26.3.412

[B53] Smith D. Wright C. J. Cantwell C. (2008). Beating the bunker: the effect of PETTLEP imagery on golf bunker shot performance. Res. Q. Exerc. Sport 79, 385–391. 10.5641/193250308x13086832906111 18816950

[B54] Soohoo S. Takemoto K. Y. McCullagh P. (2001). A comparison of modeling and imagery on the performance of a motor skill. J. Sports Behav. 27, 349–367.

[B55] Ste-Marie D. M. Law B. Rymal A. M. Jenny O. Hall C. McCullagh P. (2012). Observation interventions for motor skill learning and performance—an applied model for the use of observation. Int. Rev. Sports Exerc. Psychol. 5, 145–176. 10.1080/1750984x.2012.665076

[B56] Velentzas K. Heinen T. Tenenbaum G. Schack T. (2010). Functional mental representation of volleyball routines in German youth female national players. J. Appl. Sports Psychol. 22, 474–485. 10.1080/10413200.2010.504650

[B57] Vogt S. Di Rienzo F. Collet C. Collins A. Guillot A. (2013). Multiple roles of motor imagery during action observation. Front. Hum. Neurosci. 7:807. 10.3389/fnhum.2013.00807 24324428 PMC3839009

[B58] Weeks D. L. Anderson L. P. (2000). The interaction of observational learning with overt practice: effects on motor skill learning. Acta Psychol. 104, 259–271. 10.1016/s0001-6918(00)00039-1 10900708

[B59] Wright D. J. McCormick S. A. Birks S. Loporto M. Holmes P. S. (2014). Action observation and imagery training Improve the ease with which athletes can generate imagery. J. Appl. Sports Psychol. 27, 156–170. 10.1080/10413200.2014.968294

[B60] Wulf G. Raupach M. Pfeiffer F. (2005). Self-controlled observational practice enhances learning. Res. Q. Exerc. Sport 76, 107–111. 10.1080/02701367.2005.10599266 15810775

[B61] Zabicki A. de Haas B. Zentgraf K. Stark R. Munzert J. Krüger B. (2017). Imagined and executed actions in the human motor system: testing neural similarity between execution and imagery of actions with a multivariate approach. Cereb. Cortex 27, 4523–4536. 10.1093/cercor/bhw257 27600847

[B93] Zentgraf K. Green N. Munzert J. Schack T. Tenenbaum G. Vickers J. N.. (2009). How are actions physically implemented? Prog. Brain Res. 174, 303–318. 10.1016/S0079-6123(09)01324-7 19477348

